# Successful Repair of Glaucoma Drainage Device Tube Exposure With a Corneal Patch Graft and Double-Layer Amniotic Membrane: A Case Report

**DOI:** 10.7759/cureus.98955

**Published:** 2025-12-11

**Authors:** Shew-Fei Chee, Kar-Yong Chong, Kamaruddin Haireen, Embong Zunaina, Kwang-Sheng Ng

**Affiliations:** 1 Department of Ophthalmology and Visual Science, School of Medical Sciences, Universiti Sains Malaysia, Kota Bharu, MYS; 2 Department of Ophthalmology, Hospital Selayang, Batu Caves, MYS; 3 Department of Ophthalmology, Hospital Raja Permaisuri Bainun, Ipoh, MYS

**Keywords:** amniotic membrane, corneal patch graft, glaucoma, glaucoma drainage device (gdd), tube erosion, tube exposure

## Abstract

Tube exposure is a recognised complication of glaucoma drainage devices (GDD). We report a case of successful repair of GDD tube exposure using a corneal patch graft and double-layer amniotic membrane in a visually precious eye with previous multiple surgeries. A 47-year-old man with juvenile open-angle glaucoma (JOAG) underwent trabeculectomy in both eyes (OU) and two GDD implantations in the right eye (OD). The left eye (OS) trabeculectomy failed, leading to panophthalmitis and evisceration. Six years after the second GDD in the OD, the superonasal tube became exposed (3.8 mm). Initial repair with an amniotic membrane transplant (AMT) failed, leaving the tube partially exposed (3.2 mm) with scarred, stiff conjunctiva but no leakage. Visual acuity (VA) was counting fingers, and intraocular pressure (IOP) was 11 mmHg. Conservative management included autologous serum eye drops, topical antibiotics, oral doxycycline, and a bandage contact lens. After six months, exposure was reduced to 1.0 mm but increased to 2.0 mm by month eight. The patient underwent surgical repair with a corneal patch graft and double-layer AMT. At six months postoperatively, the tube remained fully covered with no conjunctival erosion. Tube exposure is sight-threatening and may lead to endophthalmitis; persistent or recurrent cases can be effectively managed with corneal patch grafting combined with double-layer AMT, providing durable coverage, promoting re-epithelialisation, and allowing assessment of the underlying device.

## Introduction

A glaucoma drainage device (GDD) is used to reduce intraocular pressure (IOP) in patients with glaucoma by diverting aqueous humour to an external reservoir. It may be employed as a primary treatment option in patients with a history of previous ocular surgery or trauma and in those with neovascular or uveitic glaucoma, as well as in cases where medical therapy, laser treatment, or conventional filtering surgery has failed [1].

One of the most feared complications of GDD is tube exposure, which carries a significant risk of endophthalmitis. Approximately one-sixth of affected patients develop intraocular infection following tube exposure [2]. Reported risk factors for GDD exposure include younger age, inflammation, previous ocular surgery, and female sex [3, 4].

Corneal patch graft repair is considered an effective method for managing exposed GDD, as it provides stable conjunctival coverage. Nearly 90% of eyes show no re-exposure, epithelial breakdown over the graft, scleral thinning, or ocular infection during follow-up [5]. Amniotic membrane transplantation (AMT) may additionally promote re-epithelialisation, suppress inflammation, and reduce scarring, and is particularly useful in eyes that have undergone multiple previous surgical procedures [6]. We present a challenging case of an exposed GDD tube in a visually precious eye and describe the multimodal approach used to achieve successful repair.

## Case presentation

A 47-year-old man with no systemic comorbidities was diagnosed with juvenile open-angle glaucoma (JOAG) in both eyes (OU) at the age of 22. At presentation, visual acuity (VA) was 6/9 in the right eye (OD) and 6/12 in the left eye (OS). Gonioscopy revealed open angles OU, with glaucomatous optic nerve changes, including a cup-disc ratio (CDR) of 0.7 and bayonetting. IOP measured 38 mmHg OD and 40 mmHg OS. He was commenced on triple topical IOP-lowering therapy, achieving initial control (15-19 mmHg OU). Despite advice to undergo filtering surgery, he declined and subsequently defaulted on follow-up for three years, intermittently using over-the-counter eye drops with inconsistent compliance.

Upon re-presentation, he reported progressive visual deterioration. VA had worsened to 6/48 OD and 6/60 OS, with CDRs of 0.9 (pink) OD and 0.9 (pale) OS. IOP was 36 mmHg OD and 43 mmHg OS. He was recommended four topical agents and later consented to trabeculectomy in both eyes, with the procedures performed one month apart.

Postoperatively, the left eye developed panophthalmitis and progressed to no-light-perception vision, necessitating evisceration six months after the operation. The right eye experienced bleb failure two years later, and a Baerveldt GDD was implanted. Postoperative IOP ranged between 11 and 13 mmHg on a single topical agent. The GDD functioned for eight years before IOP increased, accompanied by visual field progression, prompting implantation of a second GDD. Subsequently, he remained well-controlled on triple topical therapy.

**Figure 1:**
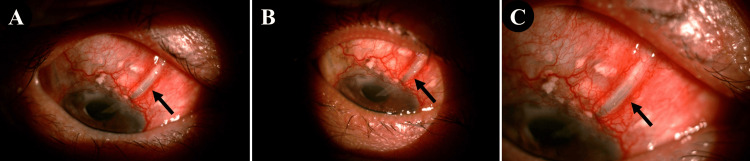
Exposed superonasal glaucoma drainage device tube in the right eye (black arrows). A: Tube exposure measuring 3.2 mm following failed amniotic membrane transplantation; B: Reduction of tube exposure to 1.0 mm at the six-month review; C: Increase in tube exposure to 2.0 mm at the eight-month review.

Six years later, routine examination revealed exposure of the superonasal GDD tube, measuring 3.8 mm. VA remained at counting fingers at 3 feet, and IOP was 11 mmHg. There was no leakage (negative Seidel’s test) or intraocular infection. Initial repair with AMT failed, leaving persistent exposure (3.2 mm) with surrounding conjunctival scarring (Figure 1A). Conservative management included autologous serum eyedrops, topical antibiotics, oral doxycycline, and a bandage contact lens while awaiting a customised scleral lens. The scleral lens eventually provided improved coverage, reducing tube exposure to 1.0 mm over six months (Figure 1B). However, by the eighth month, exposure had increased to 2.0 mm, with IOP stable and no signs of infection (Figure 1C).

**Figure 2:**
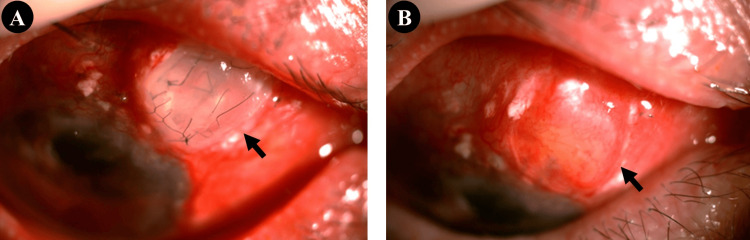
Postoperative appearance following corneal patch graft repair of the exposed superonasal glaucoma drainage device tube in the right eye (black arrows). A: Complete coverage of the tube by the corneal patch graft on day 1 postoperatively; B: Full re-epithelialisation at eight weeks postoperatively.

Given the progressive defect in his only functional eye, surgical repair was undertaken using a corneal patch graft combined with double-layer AMT. On day 1, the tube was fully covered, and complete re-epithelialisation was achieved by eight weeks (Figures 2A, 2B). At 12 months postoperatively, there was no recurrence of tube exposure or conjunctival erosion, and IOP remained stable.

## Discussion

Glaucoma is a major global public health concern, with prevalence rising in parallel with an ageing population. It is the second leading cause of blindness worldwide after cataracts and the leading cause of irreversible vision loss [7]. GDD was traditionally reserved for refractory glaucoma, whereas trabeculectomy has long been considered the surgical gold standard. However, advances in surgical techniques and expanding evidence have led to wider uptake of GDD as a primary surgical option. The Tube Versus Trabeculectomy (TVT) study demonstrated comparable outcomes and intraocular pressure reduction between GDD implantation and trabeculectomy [8].

Postoperative complications associated with GDD include hypotony, fibrous encapsulation, infection, corneal decompensation, and tube-related problems such as erosion and exposure [1]. Tube erosion is of particular concern, as it provides a potential entry point for infection and may lead to endophthalmitis. In the Ahmed Baerveldt Comparison (ABC) Study, the five-year incidence of tube erosion was 1% in the Ahmed Glaucoma Valve group and 3% in the Baerveldt implant group [9].

Several factors have been associated with an increased risk of tube erosion. Chaku et al. reported a higher prevalence of ocular inflammation in patients with tube exposure compared with controls, suggesting that immune-mediated inflammation may contribute to graft or conjunctival melting. They also identified younger age as a potential risk factor, although Al-Beishri et al. reported the opposite association. Additional reported risk factors include previous ocular surgery and female sex [3, 4].

The implantation site of a GDD must be selected carefully, as it can influence the risk of subsequent tube exposure. Inferiorly placed devices have a higher likelihood of exposure than those implanted superiorly [10]. This may be attributed to physiological pooling of the tear film inferiorly, which can harbour microorganisms, and to anatomical factors, as the inferior fornix is shorter than the superior fornix and provides less tissue for adequate implant coverage. Although the GDD was implanted in the superior fornix, the patient’s relatively young age at first implantation (late 20s) and his multiple prior surgeries may have contributed to the subsequent tube exposure.

Although primary closure by directly suturing the conjunctiva over the tube may seem appealing, it carries a high risk of failure and re-exposure. Repair of tube erosion requires careful planning. Surgery typically begins with dissection of the surrounding conjunctiva, which is often friable and non-viable. This tissue must be excised, and the dissection extended to healthy, mobile conjunctiva to allow a tension-free closure. The exposed tube is then covered with a patch graft, followed by conjunctival coverage. When conjunctival scarring limits available tissue, alternatives such as autologous conjunctival grafts, rotational pedicle flaps, amniotic membrane, or buccal mucous membrane grafts may be used to achieve adequate coverage [11, 12].

Corneal patch grafts have been used to protect GDD tubes from erosion and to repair leaking blebs following trabeculectomy [13]. Amniotic membrane is also widely employed for the repair of conjunctival defects, leaking blebs, and tube erosion, with reported success rates of up to 93% in eyes treated with amniotic membrane patch grafts [14].

The main challenges in this case were the initial failure of the AMT and significant conjunctival stiffening. At presentation, there was no leakage or evidence of endophthalmitis. Conservative management was initiated, including oral doxycycline and autologous serum eye drops, to promote epithelial healing. Although the defect initially decreased in size, it subsequently enlarged. Lagnado et al. have described the use of autologous serum drops for corneal surface disease and persistent epithelial defects, noting that their efficacy is likely related to the high concentration of growth factors that support epithelial regeneration [15]. 

We proceeded with repair using a corneal patch graft combined with a double-layer AMT. A 3 × 3 mm corneal patch graft was placed over the tube and secured with 9-0 nylon sutures. A double-layer AMT was then positioned over the patch graft, with the membrane edges tucked beneath the surrounding conjunctiva and anchored using 10-0 nylon. Corneal patch grafts provide substantial lamellar tissue with enhanced biomechanical stability and reduced susceptibility to tissue breakdown and infection [5]. The amniotic membrane also facilitates the process of re-epithelialisation [6]. The patient remains under follow-up, and this approach has thus far provided stable and effective tube coverage. A case series describing the repair of GDD tube exposure using a patch graft in combination with a double-layer AMT and adjunctive autologous serum drops is comparable to our case, with the main difference being the choice of patch graft material, sclera in their study versus cornea in ours [16]. Several studies employing different techniques for the repair of GDD tube exposure, along with their reported outcomes, are summarised in Table 1.

**Table 1:** Overview of surgical techniques employed in the management of glaucoma drainage device tube exposure and associated clinical outcomes. AMT: amniotic membrane transplant

Study	Graft Material Used	AMT	Sample Size	Outcome
Singh et al. [5]	Corneal patch graft	No	Eight eyes	Seven achieved complete closure; one required a second graft for closure
Ainsworth et al. [6]	No	Double-layer AMT	Three eyes	All cases achieved closure
Koay et al. [16]	Scleral patch graft	Double-layer AMT	Three eyes	One achieved closure; one required repeat grafting for closure; one showed late failure after four years

## Conclusions

Tube exposure is a serious, potentially sight-threatening complication following GDD implantation, with a significant risk of endophthalmitis if not promptly addressed. Conservative measures may be attempted initially, but persistent or recurrent exposure typically necessitates surgical repair. Our case demonstrates that corneal patch grafting combined with double-layer AMT provides dense, durable coverage while promoting re-epithelialisation and permitting visualisation of the underlying device. This multimodal approach offers stable, long-term protection and can be effective even in eyes with prior unsuccessful repairs. Patients who are younger at the time of implantation or have a history of multiple ocular surgeries may be at increased risk of tube exposure, underscoring the importance of careful postoperative monitoring and timely intervention.
